# Characteristics of Oral Abnormalities in Liver Transplant Candidates

**Published:** 2010-08-01

**Authors:** J. Guggenheimer, J. M. Close, B. Eghtesad, C. Shay

**Affiliations:** 1 *Department of Diagnostic Sciences, University of Pittsburgh School of Dental Medicine,*; 2 *Department of Dental Public Health and Information Management, University of Pittsburgh, School of Dental Medicine, *; 3 *Liver Transplant Center, Cleveland Clinic, Cleveland, OH, USA*

**Keywords:** Liver diseases, Mouth mucosa, Oral pathology, Xerostomia, Candidiasis

## Abstract

Background: Several oral mucosal abnormalities have been reported to occur more frequently in patients with liver disease. It has, however, not been determined if these conditions are related to the disease or are manifestations of extraneous factors not associated with the liver pathology.

Objective: To identify and quantify oral abnormalities in candidates for liver transplantation, and to determine whether these conditions were correlated with the type of liver disease or were the result of other patient variables.

Methods: Oral examinations were performed on 300 candidates for liver transplantation to assess their oral health and to record the presence and types of oral mucosal pathologies. Abnormalities most frequently encountered were analyzed for significant associations with classification of liver disease, hyposalivation, diuretic therapy, edentulism, or smoking.

Results: Among these subjects, 175 (58%) had one or more abnormalities. The anomalies most frequently found were fissured tongue (37%), atrophy of the papillae of the tongue (18%), angular cheilitis (4%) and manifestations of clinical candidiasis (2%). Clinical hyposalivation was found in 28.7% of all patients and 70% of those who were on diuretic therapy. Fissured tongue and atrophy of the tongue papillae were significantly associated with hyposalivation (p<0.001); hyposalivation was correlated to diuretic therapy (p=0.028). Pathologies suggestive of candidiasis were significantly associated with hyposalivation and total edentulism.

Conclusion: Several oral mucosal abnormalities that have previously been linked with liver diseases were found to be primarily associated with diuretic-induced hyposalivation, smoking, and total edentulism.

## INTRODUCTION

A number of oral disorders have been described in patients with liver disease. These include lichen planus, xerostomia and primary Sjögren’s syndrome which have been associated with hepatitis C [1-5]. Primary Sjögren’s syndrome has also been attributed to primary biliary cirrhosis, chronic active hepatitis, cryptogenic cirrhosis, and autoimmune hepatitis [6,7]. In addition, atrophic glossitis has been reported in patients with alcoholic hepatitis [8], and fissured tongue in liver transplant recipients [9]. The occurrence of these conditions in conjunction with liver disease may be causally related, coincidental, secondary to therapeutic interventions, or attributable to other factors that patients with advanced liver disease may have in common.

In a previous study that evaluated the dental health status of a population of potential candidates for liver transplantation, we observed that a number of the subjects had manifestations of hyposalivation, were on diuretic therapy, smoked, and were edentulous [10]. During that study, the presence of oral mucosal pathologies was also recorded. In the present report we describe the types of abnormalities we identified. To determine the pathogenesis of these lesions, they were assessed for possible associations with type of liver disease, hyposalivation, effects of diuretic therapy, smoking, and total edentulism.

## PATIENTS AND METHODS

Between 2004 and 2005, 300 consecutive patients who were being evaluated for possible liver transplantation at the University of Pittsburgh’s Starzl Transplant Institute underwent an oral examination to identify any dental treatment needs. This study had been approved by the University’s Institutional Review Board (IRB) which included patient permission to allow all medical record information to be placed in a Transplant Research Registry.

Assessments

The presence of oral mucosal abnormalities was determined by an experienced dental practitioner [JG]. The data were entered on a checklist of common oral lesions that had previously been developed to determine the prevalence of oral pathologies in a diabetic population [11]. In addition, subjective complaints of xerostomia, clinical manifestations of hyposalivation, and use of diuretic drugs were recorded. Symptoms of xerostomia were rated on a scale of 0-4 based on the number of affirmative responses to the following questions: “Does your mouth usually feel dry?” “Do you have difficulty swallowing dry foods?” “Do you have to drink liquids to help you swallow dry foods?” and “Do you feel you have enough saliva in your mouth?” [12] Dry mouth secondary to hyposalivation was determined by the visual appearance of the oral tissues and palpation of the oral mucosa. This information, along with patient demographics, liver disease diagnoses, current smoking status, and being totally edentulous was coded in order to de-identify the subjects and entered in a spreadsheet program (Microsoft Excel V. II. 3, Redmond, WA, USA).

Data analysis

The data were imported from the MS-Excel spreadsheet into SPSS (SPSS for Windows, ver 14, SPSS Inc, Chicago, IL, USA) and analyzed for statistically significant differences in proportions or means depending on the measurement level of each dependent variable. χ^2^, one-way ANOVA, and *Student’s t* test for independent samples were used for these analyses. The prevalence of more frequently encountered abnormalities and manifestations of xerostomia were also compared with data from two population studies [13,14] using a two-sample χ^2^ test for differences in proportions.

## RESULTS

**Figure 1:**
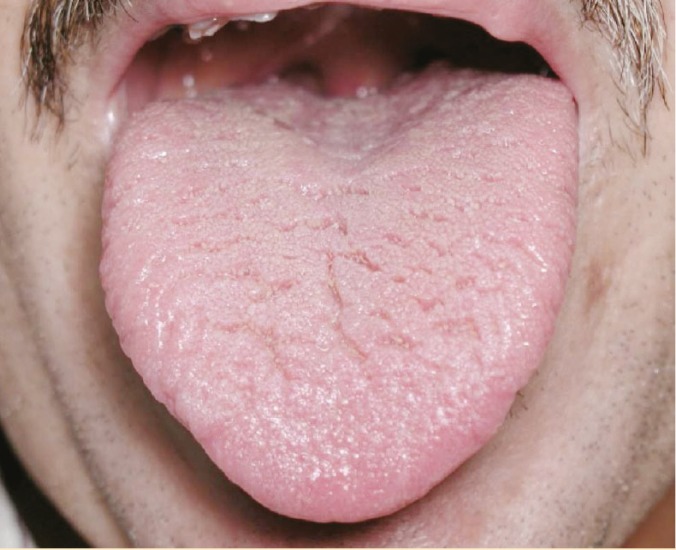
Fissured tongue in a patient with hypo-salivation

**Figure 2:**
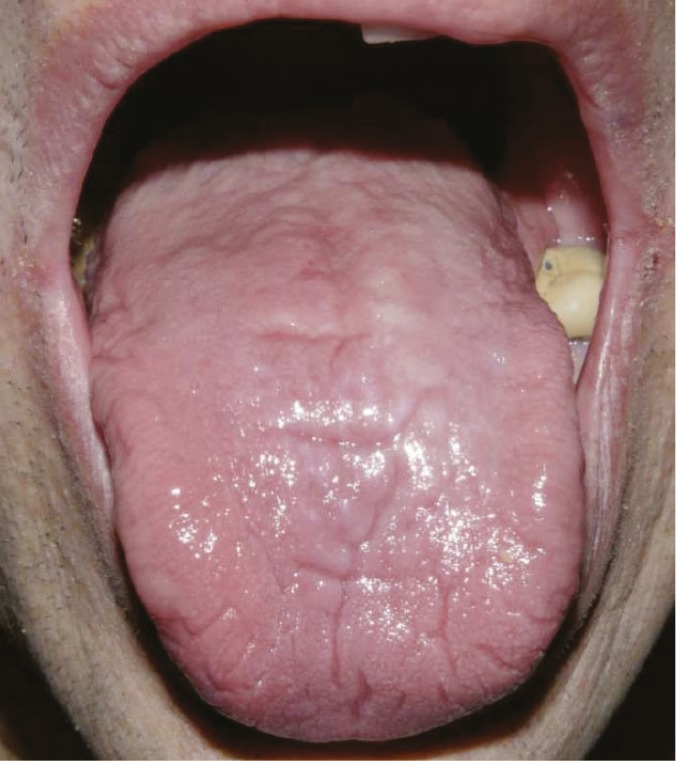
Dorsal atrophy of the tongue (atrophic glossitis). The patient was xerostomic and smoker

The demographic characteristics of the study subjects and their primary and secondary liver diseases diagnosed are shown in Tables 1a, and 1b. Patients had a mean age of 54.4 years. A secondary liver disease was present in 70 of the patients. There were 113 (37.7%) current smokers; 67 (22.3%) patients were totally edentulous, and 211 (70.3%) were taking a diuretic. Table 2 lists the oral abnormalities and their prevalence. Conditions most frequently encountered were fissured tongue (Fig. 1) and generalized atrophy of the filiform papillae that involved the entire dorsal surface of the tongue (Fig. 2). Angular cheilitis affected 12 (4%) subjects. Intra-oral candidiasis—characterized by atrophic or pseudomembranous lesions or denture stomatitis—was identified in seven patients (2.3%). When compared with the estimated prevalence rates in the general population, fissured tongue and manifestations of candidiasis occurred more frequently (p<0.0001) [13], while the prevalence of angular cheilitis, was not significantly different (p=0.170).

**Table 1a:** Demographic characteristics of the study sample

	N	%
Male	173	57.7
Female	127	42.3
Age distribution		
<50	96	32.0
50-64	144	48.0
≥65	60	20.0

**Table 1b:** Primary and secondary liver diseases diagnosed in the study sample

Liver disease diagnosis	Primary (N)	%	Secondary (N)
Hepatitis C	90	30.0	17
Alcoholic cirrhosis	80	26.7	12
Post necrotic cirrhosis, cryptogenic	41	13.7	1
Non-alcoholic steatohepatitis	26	8.7	5
Primary sclerosing cholangitis	14	4.7	0
Autoimmune hepatitis	14	4.7	1
Hepatocellular carcinoma	7	2.3	11
Primary biliary cirrhosis	6	2.0	2
Hemochromatosis	5	1.7	5
Hepatitis B	5	1.7	2
Cirrhosis, NOS*	4	1.3	3
Amyloid; sarcoid	2	0.66	0
HIV†	0	0.0	10
Other	6	2.0	1

* Not otherwise specified

† Human immunodeficiency virus infection was a secondary contributor to liver disease

**Table 2:** Oral pathologies and their potential risk factors

Pathology	Risk factor	p value
Fissured tongue	clinical hyposalivation	0.001
Atrophy of tongue papillae	clinical hyposalivation	0.001
	xerostomia index > 1	0.001
	diuretic therapy	0.049
	total edentulism	0.049
Candidiasis	xerostomia index ≥ 2	0.002
	clinical hyposalivation	0.022
Angular cheilitis	total edentulism	0.006
Angular cheilitis and atrophy of tongue papillae	total edentulism	0.001
Clinical hyposalivation	diuretic therapy	0.028

Lesions suspected to be lichen planus but which were not confirmed histopathologically, were identified in three patients. Among these cases, two (1.7%) were found in the 117 subjects with hepatitis C; the third one occurred in a patient with cryptogenic cirrhosis.

Table 2 lists the most frequently encountered pathologies for which significant associations with suspected risk factors could be identified. Based on these analyses, fissured tongue was associated with clinical hyposalivation (p=0.001). Atrophy of the tongue papillae was more likely to occur in edentulous subjects (p=0.049) who had manifestations of clinical hyposalivation (p=0.001), who reported more than one symptom of xerostomia (p=0.001), and who were on diuretic therapy (p=0.049). Clinical candidiasis was associated with subjects who had clinical features of hyposalivation (p=0.022), and who also reported two or more symptoms of xerostomia (p=0.002). Finally, angular cheilitis and atrophy of the tongue papillae were more likely to occur in patients who were totally edentulous (p=0.001).

The prevalence of subjects with symptoms of xerostomia and clinical manifestations of hyposalivation is shown in Table 2. Clinical evidence of hyposalivation was significantly associated only with diuretic therapy (p=0.028, Table 2). Neither symptoms of xerostomia nor clinical signs of hyposalivation appeared to have significant relationships with hepatitis C, HIV, or autoimmune-related liver disease. Furthermore, among the subjects with these liver diseases, no significant differences were found on the quantitative (Fox) xerostomia scale [12] based on ANOVA and controlling for diuretic therapy.

The prevalence of xerostomic symptoms in this study’s subjects was compared with that of a Swedish adult population who were on medications [14]. This comparison showed that the patients with liver disease who were between the ages 40 and 59 were significantly (p<0.05) more likely to report symptoms of xerostomia, but these symptoms were not significantly related to diuretic therapy.

## DISCUSSION

Oral pathologies that have been attributed to or associated with various liver diseases may be related to those diseases, but these abnormalities may also be manifestations of extraneous factors such as concurrent treatment modalities, patient attributes including smoking or edentulism, as well as the presence of coexisting conditions, particularly diabetes. In the present study of 300 subjects with advanced liver disease, it was determined that the most prevalent oral pathologies—fissured tongue, atrophy of the tongue papillae, angular cheilitis, and clinical signs of candidiasis—were all associated with clinical hyposalivation which was manifested by 28.7% of the subjects. Hyposalivation, in turn, was significantly correlated with the use of diuretics that were being taken by 70% of the subjects. Diuretic therapy in patients with liver disease is frequently required for the control of ascites. Ascites has been characterized as “the most common complication of cirrhosis,” and the development of ascites is often indicative of the need for liver transplantation [15]. The two classes of diuretic agents—thiazides and loop diuretics—are often used to manage cirrhosis-associated ascites [16], and both these agents have been shown to negatively affect salivary flow [17].

Atrophy of the tongue papillae or atrophic glossitis has been attributed to vitamin, iron or a combination of nutritional deficiencies for development of which those with alcoholic cirrhosis are at a greater risk [8]. In the current study, 23 (42%) of the 55 subjects with atrophic glossitis had alcoholic cirrhosis. Alcohol abuse may also be associated with hepatitis C and carcinoma of the liver. Therefore, the data were tested for the possibility of a significant relationship between atrophic glossitis and all cases of hepatitis C, carcinoma of the liver, as well as alcoholic cirrhosis (n=148), but found that the relation was not significant (p=0.115). It was determined, however, that atrophy of the lingual papillae was significantly associated with clinical hyposalivation secondary to diuretic therapy. Atrophy of the tongue papillae in conjunction with angular cheilitis also showed significant association with total edentulism which could be indicative of candidiasis. Although the presence of *Candida *hyphae was not determined, candidiasis is more prevalent with denture wearing, and can be exacerbated by hyposalivation as well as smoking [18,19]. Among the subjects with a clinical manifestation of candidiasis, 38% were current smokers, and 22% were totally edentulous.

A possible association between lichen planus and hepatitis C has been described in several reports [1,2]. The rate of suspected cases of lichen planus among the subjects in the present study (1%) was significantly greater than that found in a general population (p<0.005) [13], but was also within the estimated prevalence rates for this condition in the general population in other surveys, ranging from 0.1% to 4% [2,20]. The 1.7% rate of lichen planus among subjects with hepatitis C also remained within the estimated rate among individuals in the population who do not have hepatitis C [19,20]. Due to the small number of cases we studied, however, it could not be determined if lichen planus had any significant relationship with hepatitis C.

Among these 300 subjects, 172 (57.3%) had hepatitis C or an autoimmune liver disease as their primary or secondary diagnosis (Table 1b). Manifestations of hyposalivation in this group were found to be significantly related only to diuretic therapy, but the coexistence of primary Sjögren’s syndrome in the subjects with these liver diseases cannot be ruled out. Only one candidate, a 67-year-old woman with autoimmune hepatitis who had all four symptoms of xerostomia, reported that she had also been diagnosed with Sjögren’s syndrome. She was not taking diuretics.

Hyposalivation and a Sjögren’s-like disorder have also been described in patients with HIV infection [21,22], but in the 10 subjects who had secondary HIV-related liver disease, no significant relationship was found with clinical hyposalivation.

Although it was determined that diuretic therapy was the primary factor associated with hyposalivation, there could have been an additive effect from 13 other classes of drugs that these subjects were taking. The drugs with xerogenic potential included α_1_ agonists, anticonvulsants, antihistamines, benzodiazepines, β_2_-adrenergic agonists, dopamine reuptake inhibitors, hypnotic non-benzodiazepines, interferons, opioids or non-opioid analgesics, phenothiazines, proton pump inhibitors, and serotonin reuptake inhibitors [23]. Hyposalivation as a consequence of mood disorders, independent of their treatment, represents another possible confounding factor that has been reported [24]. Liver transplant candidates are likely to be confronted with multiple sources of anxiety, stress and/or depression. These include disability, unemployment and financial insecurity, physical discomforts, whether or not the criteria for transplantation will be met, followed by an indeterminate interval on the transplant waiting list for a suitable donor organ. Between 2003 and 2004, the median time on the waiting list for liver transplantation ranged from 9.6 to 14.7 months [25].

It is also possible that diabetes mellitus could have been related or contributed to some of the oral abnormalities in these candidates for liver transplantation. Diabetes and liver disease often occur concurrently as a consequence of either the development of the liver disease from preexisting diabetes, or the diabetes developing as a complication of liver disease, particularly in patients with chronic hepatitis C, nonalcoholic fatty liver disease, and cirrhosis [26]. In a previous study of subjects with type 1 diabetes, significantly higher prevalence rates of atrophy of the tongue papillae, fissured tongue, angular cheilitis, xerostomic symptoms, candidal lesions, and clinical hyposalivation were found [11,18,27]. The oral changes that were identified in the present study could, therefore, have also resulted from diabetes, but the presence of this condition was not determined.

Our study found a current smoking rate of 37.7% that ranged from 33.3% for the women to 50% among the men [10]. Patients with alcoholic cirrhosis and/or hepatitis C had a smoking rate of 79%. We identified three patients who had lesions consistent with frictional leukoplakia which is not considered to be a premalignant lesion. Nevertheless, the high rates of smoking in this population which usually is also older, increases the risk for the development of *de novo* malignancies, particularly in the oropharyngeal and gastrointestinal tracts [28]. Clinicians need to remain aware of the possibility of this occurrence.

## CONCLUSIONS

Several oral abnormalities that were identified more frequently in candidates for liver transplantation were found not to be related to specific liver diseases. These conditions may, instead, have resulted from changes in the oral environment that are similar to those that may be encountered in other medically compromised populations, including diabetics. The oral mucosal pathologies that were encountered could, therefore, be attributed to interactions among a number of extraneous risk factors, including use of xerogenic drugs, hyposalivation, or candidiasis.

## References

[1] Chainani-Wu N, Lozada-Nur F, Terrault N (2004). Hepatitis C virus and lichen planus: a review. Oral Surg Oral Med Oral Pathol Oral RadiolEndod.

[2] Lodi G, Scully C, Carrozzo M (2005). Current controversies in oral lichen planus: report of an intenational consensus meeting. Part 1. Viral infections and etiopathogenesis. Oral Surg Oral Med Oral Pathol Oral RadiolEndod.

[3] Cacoub P, Renou C, Rosenthal E (2000). Extrahepatic manifestations associated with hepatitis C virus infection. A prospective multicenter study of 321 patients. The GERMIVIC Medicine.

[4] Ramos-Casals M, Loustaud-Ratti V, De Vita S (2005). Sjögren syndrome associated with hepatitis C virus. A multicenter analysis of 137 cases. Medicine.

[5] Scott CA, Avellini C, Desinan L (1997). Chronic lymphocytic sialoadenitis in HCV-related chronic liver disease: comparison with Sjögren’s syndrome. Histopathology.

[6] Abraham S, Begum S, Isenberg D (2004). Hepatic manifestations of autoimmune rheumatic diseases. Ann RheumDis.

[7] Matsumoto T, Morizane T, Aoki Y (2005). Autoimmune hepatitis in primary Sjögren’s syndrome: pathological study of livers and labial salivary glands in 17 patients with primary Sjögren’s syndrome. Pathol Int.

[8] Golla K, Epstein JB, Cabay RJ (2004). Liver disease: current perspectives on medical and dental management. Oral Surg Oral Med Oral Pathol Oral Radiol Endod.

[9] Diaz-Ortiz ML, Mico-Llorens JM, Gargallo-Albiol J (2005). Dental health in liver transplant patients. Medicina Oral, Patologia Oral y Cirugia Bucal.

[10] Guggenheimer J, Eghtesad B, Close JM (2007). The dental health status of liver transplant candidates. Liver Transpl.

[11] Guggenheimer J, Moore PA, Rossie K (2007). Insulin dependent diabetes mellitus and oral soft tissue pathologies. I. Prevalence and characteristics of non-candidal lesions. Oral Surg Oral Med Oral Pathol Oral Radiol Endod.

[12] Fox PC, Busch KA, Baum BJ (1987). Subjective reports of xerostomia and objective measures of salivary gland performance. J Am Dent Assoc.

[13] Shulman JD, Beach MM, Rivera-Hildalgo F (2004). The prevalence of oral mucosal lesions in U.S. adults. Data from the third national health and nutrition examination survey, 1988-1994. J Am Dent Assoc.

[14] Nederfors T, Isaksson R, Mornstad H, Dahlof C (1997). Prevalence of perceived symptoms of dry mouth in an adult Swedish population-relation to age, sex, and pharmacotherapy. Community Dent Oral Epidemiol.

[15] Ginès P, Cárdenas A, Arroyo V, Rodès J (2004). Management of cirrhosis and ascites. N Engl J Med.

[16] Runyon BA (2004). AASLD Practice Guideline. Management of adult patients with ascites due to cirrhosis. Hepatology.

[17] Nederfors T, Nauntofte B, Twetman S (2004). Effects of furosemide and bendroflumethiazide on saliva flow rate and composition. Archiv Oral Biology.

[18] Guggenheimer J, Moore PA, Rossie K (2000). Insulin dependent diabetes mellitus and oral soft tissue pathologies. II. Prevalence and characteristics of Candida and candidal lesions. Oral Surg Oral Med Oral Pathol Oral Radiol Endod.

[19] Shimizu C, Kuriyama T, Williams DW (2008). Association of oral yeast carriage with specific host factors and altered mouth sensation. Oral Surg Oral Med Oral Pathol Oral Radiol Endod.

[20] Neville BW, Damm DD, Allen CM, Bouquot JE (2002). Oral and Maxillofacial Pathology.

[21] Schiodt M, Dodd CL, Greenspan D (1992). Natural history of HIV-associated salivary gland disease. Oral Surg Oral Med Oral Pathol.

[22] Kordossis T, Paikos S, Aroni K (1998). Prevalence of Sjogren’s-like syndrome in a cohort of HIV-1-positive patients: descriptive pathology and immunopathology. Br J Rheumatol.

[23] Wynn RL, Meiller TF, Crossley HL (2007). 2007-2008Drug Information Handbook for Dentistry.

[24] Bergdahl M, Bergdahl J (2000). Low unstimulated salivary flow and subjective oral dryness: association with medication, anxiety, depression, and stress. J Dent Res.

[25] Organ Procurement and Transplantation Network Liver Kaplan-Meier median waiting times for registrations listed: 1999-2004.

[26] Harrison SA (2006). Liver disease in patients with diabetes mellitus. J Clin Gastroenterol.

[27] Moore PA, Guggenheimer J, Etzel KR (2001). Type 1 diabetes mellitus, xerostomia, and salivary flow rates. Oral Surg Oral Med Oral Pathol Oral Radiol Endod.

[28] Herrero J (2009). De novo malignancies following liver transplantation: impact and recommendations. Liver Transpl.

